# Robust orthogonal recombination system for versatile genomic elements rearrangement in yeast *Saccharomyces Cerevisiae*

**DOI:** 10.1038/srep15249

**Published:** 2015-10-19

**Authors:** Qiuhui Lin, Hao Qi, Yi Wu, Yingjin Yuan

**Affiliations:** 1 Key Laboratory of Systems Bioengineering (Ministry of Education), Tianjin University, Tianjin, 300072, PR China; 2 SynBio Research Platform, Collaborative Innovation Center of Chemical Science and Engineering (Tianjin), School of Chemical Engineering and Technology, Tianjin University, Tianjin, 300072, PR China

## Abstract

Rearrangement of genomic DNA elements in a dynamic controlled fashion is a fundamental challenge. Site-specific DNA recombinases have been tamed as a powerful tool in genome editing. Here, we reported a DNA element rearrangement on the basis of a pairwise orthogonal recombination system which is comprised of two site-specific recombinases of Vika/vox and Cre/loxp in yeast *Saccharomyces Creevisiae*. Taking the advantage of the robust pairwise orthogonality, we showed that multi gene elements could be organized in a programmed way, in which rationally designed pattern of loxP and vox determined the final genotype after expressing corresponding recombinases. Finally, it was demonstrated that the pairwise orthogonal recombination system could be utilized to refine synthetic chromosome rearrangement and modification by loxP-mediated evolution, SCRaMbLE, in yeast cell carrying a completely synthesized chromosome III.

Genome editing is emerging as a powerful technology platform which paved the way for exploring the nature of life comprehensively and systematically^1^. In the past few years, by taking the advantage of quickly developing bio-technology significant successes have been achieved in manipulating large DNA fragment of size up to million base pairs^2^,^3^,^4^. Genome-wide manipulation such as rewiring genetic codons^5^, genome streamlining^6^,^7^, redesigning artificial chromosome^8^,^9^ become feasible and practical. Site-specific DNA recombination has been validated as one of the most powerful genetic editing tools^10^. Due to the high specificity in recognizing target DNA sequence, site-specific recombinases have been implemented in various bioengineering applications^11^. Recently a milestone was reached with achieved total synthesis of functional chromosome III DNA in yeast, *Saccharomyces Cerevisiae*^12^,^13^. This synthetic chromosome was constructed with numerous redesigns including rewired genetic codons and streamlined genome architecture. These designs were supposed to simplify chromosome and improve the ease of further engineering in the future. Furthermore, an amazing feature was constructed as well. It allows cell to evolve in a specifically controlled fashion. Particularly it is achieved via making use of an engineered site-specific DNA recombination system^14^,^15^,^16^ by which recombinase Cre will with equal chance reverse or delete targeted DNA region flanked by two loxPxym sites. Therefore, upon expressing recombinase Cre, the synthetic chromosome III will undergo a random rearrangement process, termed as SCRAMBLE, synthetic chromosome rearrangement and modification by loxP-mediated evolution^17^. Due to the presence of 98 loxP sites on the synthetic DNA structure, a huge number of random combinations in rearranging DNA elements could be expected. It has been demonstrated that recombinase mediated scrambling enable yeast cell to generate various genotypes corresponding to a broad variety of phenotypes.

Yeast is one of the well-studied cell models, which has significantly contributed to modern eukaryotic biology^18^ and also the pioneer organism, which has been utilized in food manufacturing through fermentation since the very early of human history^19^. With the emerging biotechnology, yeast is attracting more attentions in metabolic engineering applications^20^,^21^. Herein, we reported that another site-specific DNA recombinase Vika, originally identified in a gram-negative bacterium *Vibro coralliilyticus*, could functionally and specifically deleted genomic DNA fragment via recognizing specific DNA site vox in yeast *Saccharomyces cerevisiae*^22^,^23^ and other spices^24^, including mammal cell and bacteria. In spite of DNA sequence in recognizing site of loxP and vox shares a high similarity, it was demonstrated that activities of the two recombination systems were strictly independent to each other in yeast. Furthermore, the Vika-vox system functioned properly even in yeast cell carrying the synthesized chromosome III on which 98 loxPsym sites are encoded. Based on this robust pairwise orthogonal of Cre and Vika recombinases, we designed versatile genetic editing cassette by which DNA elements could be rearranged following a designed process and the final genotype output could be controlled through simply designing the pattern of loxP and vox location. As proof of concept, it was demonstrated that two targeted genes could be deleted in a timely or selectively controlled fashion in corresponding designed editing cassette.

The Cre/loxP driven genome scrambling makes the synthesized chromosome III a promising platform for metabolic engineering and efficient directed evolution. However, in the presence of the high number of loxP recombinase recognition site on the synthetic chromosome even the leak of recombinase expression resulted to the genome changing constantly. It was a critical problem in maintaining and analyzing the yeast cell screened out from the scrambled cell library. Therefore, we introduced the Vika-vox system into the SCRaMbLE of synthetic chromosome III and demonstrated that Vika-vox was able to efficiently terminate the genome scrambling through completely deletion of gene Cre. We believed that the robust orthogonal recombinase system could be powerful in developing highly controlled DNA element editing tool and especially for developing enhanced recombinase-drive genome directed evolution system.

## Results

### Construction of an orthogonal recombinases system in budding yeast cells

**Figure 1:**
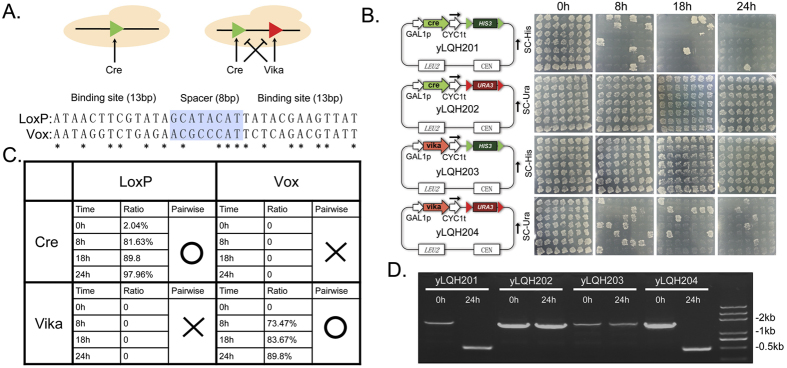
Orthogonal recombination in yeast. (**A**) The schematic of the orthogonal recombination system comprising two site-specific recombianses, Cre and Vika in yeast and their DNA recognition sequences in which homology parts were marked out with star and the 8 bp spacer parts were marked out as gray. (**B**) The pairwise between recombinase Cre, Vika and DNA recognition site of LoxyP and vox were evaluated. Strain yLQH201 was transformed with DNA encoding Cre and loxP (green triangle)-HIS3 (selection marker)-loxP; yLQH202 was transformed with DNA encoding Cre and vox (red triangle)-URA3 (selection marker)- vox; yLQH203 was transformed with DNA encoding vika and loxP (green triangle)-HIS3 (selection marker)-loxP; yLQH204 was transformed with DNA encoding vika and vox (red triangle)-URA3 (selection marker)- vox. DNA excision was induced by initiating recombinase expression and (**C**) measured by counting the colony cultured on the corresponding selective plate and further confirmed by amplifying corresponding DNA fragment, original DNA structure was amplified as 2 kb fragment and DNA structure was amplified as 0.5 kb fragment after correct gene excision (**D**).

For developing sophisticated controllability on the basis of DNA site-specific recombination to precisely regulate DNA rearrangement, we challenged to construct a pairwise orthogonal recombination system in budding yeast cell. Due to the high efficiency and site-specificity, Cre/loxP becomes the most widely used recombination system in genetic engineering. In this research, we introduced another recombination system of Vika/vox into yeast. Recombinase Vika was identified in bacteriophage of *vibrio coralliilyticus* through a broad alignment searching and proven as a high efficient recombinase in heterologous hosts including bacterial and mammalian cell^22^,^23^. Since the two systems belong to the same tyrosine recombinase family, they share high similarity in both sequence and secondary structure in their DNA recognition sites, in which one 8 base pairs spacer sequence are flanked by 13 base pairs inverted repeated sequence (Fig. 1A). Although there was no apparent cross-activity between recombinases Vika and Cre were measured in host of E. coli, mouse or human cells, the catalysis activities of these two recombinases in yeast cells which possessing efficient intrinsic homology recombination system was still not studied. Vector carrying recombinase Cre or Vika under control of inducible promoter and genetic selection marker flanked by specific recognition site of loxP or vox respectively were constructed and transformed in *Saccharomyces cerevisiae* cell. The excision of genetic marker was analyzed with growing cells on selective solid medium plate. High efficient of 81.63% and 73.47% for disruption of specific target gene marker by Cre or Vika respectively after 8 hrs of recombinase expression and 97.96% and 89.8% for 24 hrs recombinase expression were achieved (Fig. 1B,C). In contrast, no gene marker disruption was observed when Cre or Vika was expressed to excise gene marker targeted by vox or loxP sites respectively. To further confirm the gene marker disruption, the DNA vectors were isolated and its genotypes were analyzed by *in vitro* amplification (Fig. 1D). There was a clear DNA fragment of 2 kb amplified at time point 0 h in all the strains before recombinase expression. In contrast, after 24 h of recombinase expression, the amplified DNA fragment size shifted to around 0.5 kb in strain of yLQH201 and yLQH204, but no difference was observed in strain of yLQH202 and yLQH203. These results were consistent with the auxotrophy selective culture analysis and demonstrated that site-specific recombinases of Cre and Vika possess high specificity in recognizing its target DNA sequence respectively and are able to achieve their function simultaneously in yeast cell in an orthogonal manner.

### Rational designed genetic elements rearrangement

**Figure 2:**
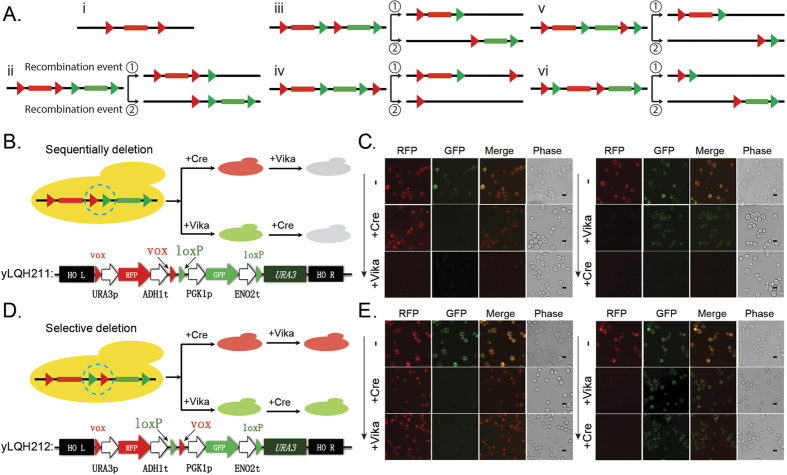
Rational designed DNA rearrangement. (**A**) Schematic of the logically controlled DNA rearrangement through designing the location of orthogonal recombinase recognition sizes. (**B**) Schematic of the designed DNA cassette for sequential deletion of two genes in yeast based on Cre/Vika orthogonal recombination system. Report gene GFP and RFP was flanked with vox and loxP respectively. (**C**) It was observed that GFP or RFP was excised accordingly when Cre or Vika was expressed firstly, and then the left report gene RFP or GFP was excised accordingly when Vika or Cre was expressed successively. (**D**) Schematic of the designed DNA cassette for selective deletion of two genes in yeast based on Cre/Vika orthogonal recombination system. Similarly, report gene GFP and RFP was flanked with vox and loxP respectively, but the location of vox and loxP site in the middle was switched. (**E**) It was observed that GFP or RFP was excised accordingly when Cre or Vika was expressed firstly, however, the left report gene RFP or GFP cannot be excised even after both of recombinases were expressed. As described in method, fluorescence and phase imaging was obtained using fluorescent microscope (Olympus CX41). The scale bar is 10 um.

The orthogonal catalysis activity between Cre/loxP and Vika/vox frame the basis for rearranging genetic elements in a designed fashion which may impossibly be achieved by either of Cre and Vika solo. Based on the nature of the DNA recombination mechanism, two specific recognition sites are necessarily required for efficient DNA recombination. Artificial recombination function could be designed from two orthogonal recombinases. With simply combinatorial utilization of orthogonal DNA recognition sites, recombination of targeted genetic elements could be dynamically controlled and the final genotype will be determined as the function of the pattern of loxP and vox on the designed DNA fragment. (Figure 2A). For instance, two genetic elements could be selectively disrupted sequentially, or selectively protect one element from the second recombination event, or selectively disrupt either two elements together or just one of them.

To prove the concept of recombinase mediated DNA rearrangement with designed function, we designed a genetic cassette carrying two fluorescent report genes of GFP and RFP flanked by loxP and vox site respectively. With simply switching the pattern of loxP and vox site located on the cassette, sequential deletion and selective deletion of the two report genes were successfully achieved in a high efficient way. In particular, in the case of GFP and RFP are flanked by two loxP sites and two vox sites respectively (Fig. 2B), either of the two genes could be disrupted. When the expression of Cre and Vika were turned on in a sequential way, it was observed that the green and red fluorescence turned off successively. Moreover, the order in which the gene got excised was precisely controlled through regulating the expression of Cre and Vika (Fig. 2C). Next, we designed a hetero recombinase recognition system consisting of one loxP and one vox to flank the report gene of GFP and RFP respectively (Fig. 2D). Particularly in this design, the DNA sequence covered by loxP and vox sites are overlapped, resulting that only one loxP and one vox site were left on the genetic cassette after the first recombination event. Being different to the first case, one of the two report genes could remained even both of the recombinase, Cre and Vika, were expressed and which gene will be disrupted also could be controlled by regulating the expression pattern of the two recombinases. Actually, it was observed that only green fluorescence turned off if Cre was induced for expression first, and only red fluorescence turned off if Vika was induced for expression first (Fig. 2E).

### Stabilization of SCRaMbLEed synthetic yeast by robust orthogonal recombinases

**Figure 3:**
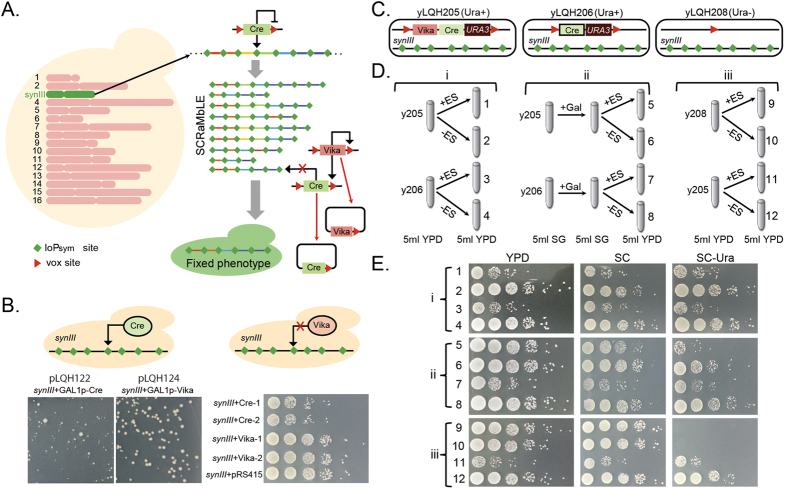
Terminate SCRaMbLEd yeast using orthogonal Cre/Vika recombination system. (**A**) The schematic of SCRaMbLE termination in synthetic yeast *synIII* by Vika mediated cre excision to obtain yeast strain with stabilized genotype and fixed phenotype. (**B**) Expression of recombinase Cre initiated SCRaMbLE in synIII and generated colonies with various morphology and size on plate (left); In contrast, expression of recombinase Vika didn’t induce any obvious change in growth of yeast synIII on plate. Schematic of genotype of strain (**C**) and assay design (**D**) for assessing Vika mediated stabilization of SCRaMbLEd synIII. Particularly, yeast synIII cells carrying yLQH205, encoding gene cre, vika and selection marker ura3, were cultured with (assay1) or without (assay2) induced Cre expression; yeast synIII cells carrying yLQH206, encoding gene cre and selection marker ura3, were cultured with (assay 3) or without (assay 4) induced Cre expression; After Vika was induced for expression, yeast synIII cells carrying yLQH205 were cultured with (assay 5) or without (assay 6) induced Cre expression and yeast synIII cells carrying yLQH206 were cultured with (assay 7) or without (assay 8) induced Cre expression; Yeast synIII cells carrying yLQH208, empty vector as control, were cultured with (assay 9) or without (assay 10); Assay 11 and assay 12 is same to assay 1 and assay 2 respectively; (**E**) SCRaMbLE of yeasts and termination of scrambled yeast cells was evaluated by measuring its growth on solid medium plate.

A modified loxP site, termed as loxPsym, with symmetrical sequence structure is capable of directing the recombination process in a non-directional way to invert or delete the targeted DNA fragments with equal possible probability^25^. On the basis of its unique properties in rearranging DNA elements, a novel genetic evolution technology has been developed, in which genetic elements were rearranged in a random way. In particular, the DNA fragment flanked by two loxPsym sites will be randomly repeated into multi-copies, inverted to opposite direction, removed to another location, or deleted to generate a huge genomic diversity in a short period. Furthermore, successful artificial synthesis of whole yeast chromosome improved this technology for the whole genome evolution, namely, synthetic chromosome rearrangement and modification by loxP-meidated eveolution. However, because quickly random changing on genome is lethal, the expression of recombinase Cre has to be controlled strictly. Here, we demonstrated that the utilization of orthogonal Cre/Vika system could be utilized to terminate the SCRaMbLE process resulting the fixed phenotype in a synthetic *Saccharomyces cerevisiae* strain depicted as yeast synIII which carrying a complete synthesized chromosome III^13^. In particular, 98 loxPsym sites were incorporated on chromosome III to target nonessential genetic elements and SCRaMbLE process will be initiated by expression of recombinase Cre from a designed DNA cassette in which gene cre and vika are both marked by vox site. Upon expression of recombinase Vika, SCRaMbLE will be terminated completely because the disruption of gene cre and vika, and cell of interesting phenotype could be screened out from the pool of cells with various but stable genotypes (Fig. 3A).

First, we assessed the specificity of activity of recombinase Vika in yeast synIII. Vector pLQH122 carrying gene vika and vector pLQH124 carrying gene cre were transformed to yeast synIII respectively and recombinases were expressed after GAL was supplemented. Distinct colony morphologies were observed between yeast synIII cells after growth on Ura selective agarose plate for 3 days. Due to the Cre mediated SCRaMbLE, cell of yeast synIII carrying pLQH122 mostly formed uneven small size except only a few of colonies with normal size. On the contrary, cell of yeast synIII carrying pLQH124 grown normally and formed relatively uniform colony with general size on solid medium plate after 3 days culture. Also, the cell growth was analyzed by plating random selected colony on solid medium as 10-fold serial dilutions. The expression of recombinase Cre slowed down the cell growth. By comparison, cells expressing recombinase Vika grew as quick as the control sample of cells which only carrying an empty vector without expressing either of recombinase Cre or Vika (Fig. 3B). Furthermore, recombinase Vika was measured with a high efficiency of about 93.9% in excising DNA fragment encoding itself (Fig. S1). These experiments successfully demonstrated the robust specificity of reombinase Vika without cross recognizing loxPsym sites even existing in abundance on the genome. After confirming the orthogonality between Vika and loxPsym, we examined the utilization of recombinase Vika to terminate the Cre mediated SCRaMbLE obtaining strain with stable genome for analysis. Vectors carrying both of gene cre and vika flanked by vox sites, or only gene of cre flanked by vox sites, or nothing as control were constructed and depicted as yLQH205, yLQH206 or yLQH208 respectively (Fig. 3C). In a first group of experiments, yLQH205 and yLQH206 was transformed to yeast synIII and initiated the SCRaMbLE, which were measured by comparing the cell growth on solid medium. The cell growth decreased significantly with the expression of Cre. However, in the second group of experiments, SCRaMbLE was clearly terminated (cell growth of assay 7 decreased one order of magnitude in comparison with assay 5) and cell growth was restored only in yeast synIII transformed with yLQH205 not yLQH206 following the expression of recombinase Vika prior to the expression of Cre (Fig. 3D,E). However, after expression of Vika, cells carrying yLQH205 still exhibited growth on SC-Ura solid media. It is probably due to cells with very high density were used to inoculate for growth on solid media and residual enzyme of URA3 from leaked expression caused.

## Discussion

Site-specific recombination is comprised of the essential part of genome manipulation technology. Due to the relatively simple mechanism of recognizing target DNA with specific sequence, recombinases are widely recruited in rearrangement of genetic elements *in vivo*. However, bioengineering applications with increasing ambition require more efficient and sophisticated genomic manipulation tools. In this study, we demonstrated a robust pairwise orthogonal recombination system in budding yeast. In this system, site-specific recombinase Vika was recruited to pair up with the recombinase Cre. Both of the two recombinases achieved its function efficiently without obviously cross reading other DNA recognition site, even there is very high similarity in the sequence and secondary structure of loxP and vox (Fig. 1). Therefore, the pairwise orthogonality could be ascribed to the high specificity in activity of Cre and Vika. To the best of our knowledge, this is the first research reporting the site-specific recombination function of Vika-vox and the orthogonality of DNA recombination activity between Cre and Vika in yeast *Saccharomyces cerevisiae*, a crucial model for eukaryotic biology and fundamental platform for metabolic engineering.

The orthogonal recombination system is a promising tool for genetic manipulation with great potential in developing novel function. Except the basic function of excising DNA for recombination, there is limited thing is possible for only one recombinase. However, new function could be created when using two orthogonal recombinases. We presented that DNA element rearrangement function with distinct logic controllability was built through rationally designing simple combination of loxP and vox site. Particularly, DNA cassette in which genes flanked between loxp and vox site respectively could remove target gene one by one. However, with simply switching the location of the two adjacent loxP and vox the DNA cassette is able to selectively delete one target gene and leave another safe no matter if expressing both recombinases. The final genotype is determined by the pattern of loxP and vox on the cassette. Moreover, it is obvious that more patterns could be designed to rationally program the rearrangement of DNA elements (Fig. 2A). This programmability developed from the pairwise orthogonal recombinases fit the requirement of application which need dynamic controllability on multiple DNA elements.

SCRaMbLE, synthetic chromosome rearrangement and modification by loxP-meidated evolution, is a significant platform for engineered yeast genomic evolution for combinatorial mutagnesis and massive restructuring of yeast genome. However, chromosomal mutation causes lethality. Due to the high activity of recombinase Cre, it was observed that even a little bit of leak in Cre expression will be efficient in turning on the SCRaMbLE process (Data not published). As known, it is difficulty to strictly suppress the gene expression from special promoter in the conventional inducible gene expression system without desired inducing. The Cre/Vika orthogonal recombinases could be recruited as platform to achieve strict controllability on gene expression via completely silencing the target gene. In comparison with the conventional system, in which special DNA elements are utilized to control targeted gene expression, technically orthogonal recombinases possess great superiority in controlled expression of toxic gene.

Technically, it is difficult to maintain a strain with unstable genome, and will result inconsistence in the long-term study. However, efficient evolution experiment has to be performed under strong expression of Cre for running SCRaMbLE in a short period and then switching it off strictly. Herein, we demonstrated that recombinase Vika could be used to excise both of recombinase genes efficiently and completely to terminate SCRaMbLE. This is a robust system in which recombinase Vika did not interfere with the growth of yeast synIII encoding a large number of 98 loxPsym sites on its synthesized chromosome III. These results demonstrated that pairwise orthogonal recombinase Vika/Cre could be an efficient solution to improve SCRaMbLE generating genetically stable yeast strain. Moreover, we believe that new genetic design principle for recombinase mediated genome evolution could be developed with taking advantage of the pairwise orthogonal Cre/Vika recombination system and combining with other DNA manipulation system.

## Materials and Methods

### Plasmids and Strains

All the yeast strains used in this study are listed in supplementary Table S1. BY4741 (MATa Leu2∆0 LYS2 met15 his3∆1 ura3∆0), BY4742 MATα (his3△1, leu△0, lys2△0, ura3△0) and *synIII* (MATα MET15 lys1∆0 ura3∆0 his3∆1leu2∆0 synIII sup61::HO) were gift from Prof. Jef Boeke (The Johns Hopkins University, USA). Unless specially mentioned, yeast culture was performed according to the yeast protocol handbook (Clontech).

Vectors including pRS413, pRS414, pRS415, pRS416 and pLM158 were gifts from Prof. Jef Boeke (The Johns Hopkins University, USA). Gene vika with optimized codon for expression in yeast was synthesized by Genewiz, Inc. and cloned into pUC57 vector as pUC57-Vika. Gene vika and cre were amplified from pUC57-Vika and pLM158 before cloned into plasmid pYES2 between *Hind*III and *Xba*I sites to construct pLQH122 (pYES2-GAL1p-Cre) and pLQH124 (pYES2-GAL1p-Vika). Gibson assembly was performed to construct Cre and Vika genes into pRS415 vector following a standard protocol. Briefly, fragment of vika and cre with GAL1 promoter were amplified from pLQH122 and pLQH124 and then assembled into *EcoRI*-digested pRS415 as pLQH134 (pRS415-pGAL1p-Cre-CYC1t) and pLQH135 (pRS415-pGAL1p-Vika-CYC1t) respectively.

Expression fragments for cre and vika were amplified from pLQH122 and pLQH124 using primers (Supplementary Table S2) and then connected with fragments of vox-*URA3-*vox and loxP-*HIS3*-loxP amplified from pRS416 and pRS413 respectively via homology assembly in yeast, the strain was denoted as yLQH201, yLQH202, yLQH203 and yLQH204.

### Recombination assay in yeast

Single colony was picked up and cultured overnight in 2% glucose SC-LEU-HIS or SC-LEU-Ura liquid medium and then transferred to 2% galactose SG-LEU medium to initiate recombinase expression. At time point of 0 h, 8 h, 18 h and 24 h after recombinase expression, cells were washed twice to remove galactose and then plated on SC-LEU plates for overnight. 49 colonies were randomly picked up to a new SC-LEU plates and replicate to either SC-Ura or SC-His plates. Success recombination was measured by counting the number of colonies with auxotrophy on each plate. Mean values were calculated from three separated experiments to quantify the efficiency of special recombination event.

### Sequential and selective deletion assay

DNA fragment of RFP and GFP were cloned into vector pLQH94 encoding a URA3 selection marker and were flanked by two HO sequences. The deletion cassettes were then inserted into the genome on HO locus and obtained strains yLQH211 (MATa Leu2∆0 LYS2 met15 his3∆1 ura3∆0 HO::vox-RFP-vox-loxP-GFP-loxP-URA3) and yLQH212 (MATa Leu2∆0 LYS2 met15 his3∆ ura3∆0 HO::vox-RFP-loxP-vox-GFP-loxP-URA3). Expression of Cre was induced after transforming the plasmid pLM158 into yeast strains yLQH211 and yLQH212 and selected on SC-His plates. Isolated colonies were inoculated overnight in 5 mL SC-His and then treated in SC-His plus estrodial medium for 12 h. Cultures with volume of 200 μL were plated on YPD plates overnight to avoid residual fluorescence. The RFP and GFP fluorescence were then imaged from this cell lawn using fluorescent microscope (Olympus CX41) In this study, Cre-EBD on pLM158 was all induced by re-inoculated the overnight culture to an OD600 of 0.1 in medium with estrodial to a final concentration of 1 μM. Similar procedures were performed for expression of Vika, except that plasmid pLQH135 was transformed and the resulting strains were induced in 2% galactose SG-Leu for 12 h.

### Recombination effect in synthetic yeast strain *synIII*

To evaluate the growth defect after induction of Cre and Vika recombinase in synthetic strain *synIII* which harbouring 98 loxPsym sites on chromosome III, two experiments were performed. In the first assay, plasmids pLQH122 and pLQH124 with 2 μ ori were transformed into *synIII* strain and plated on SC-Ura plates, respectively. Colony morphology was imaged after 3 days of inoculation in 30 °C. In the second assay, plasmids pRS415, pLQH133 and pLQH134 were transformed and selected on SC-Leu plates, where pRS415 was used as control. Single colonies were inoculated in 5 mL SC-Leu overnight and re-inoculted to an OD600 of 1.0 in 2% galactose SG-Leu medium. Cells were inoculated for 4 h and 5 μL of 10-fold serial dilutions were spotted on SC-Leu plates. Two colonies were randomly picked up for transformants with pLQH122 and pLQH124 for parallel.

### Termination of SCRaMbLE in synthetic yeast strain *synIII*

Cre-EBD fusion proteins, with and without Vika recombinase, flanked by two vox sites were integrated into Trp locus in the *synIII* strain, respectively. Cre-EBD including SCW11 promoter was amplified from pLM158 while Vika under control of GAL1 promoter was amplified from pLQH124. Primers listed in Supplementary Table S2 were used for introducing homologous sequences to facilitate genome incorporation. After transformation, cells were plated on the SC-Ura plates and replica to SC-Trp plates to select for Ura^+^Trp^−^ colonies. The resulting yeast strains yLQH205 (MATα MET15 lys1∆0 ura3∆0 his3∆1leu2∆0 synIII sup61::HO trp1::pSCW11-Cre-pGAL1-Vika-Ura3) and yLQH206 (MATα MET15 lys1∆0 ura3∆0 his3∆1leu2∆0 synIII sup61::HO trp1::vox-pSCW11-Cre-Ura3-vox) were induced by estradiol for Cre expression and galactose for Vika expression. Yeast strains yLQH208 (MATα MET15 lys1∆0 ura3∆0 his3∆1 leu2∆0 synIII sup61::HO trp1::vox) was obtained by inducing Vika expression in yLQH205 and selected for Ura^−^ phenotype.

SCRaMbLE induction results in loss of loxPsym-flanked segments, thus the fitness of strains were used as indicator for SCRaMbLE efficiency. Yeast strains yLQH205 and yLQH206 were either induced by estradiol for 6 h directly or after inoculated in galactose for 24 h. For each SCRaMbLE experiment, strains were inoculated in the same conditions only without estradiol as control group. After induction, 5 μL of 10-fold serial dilutions were spotted on YPD, SC and SC-Ura plates, and inoculated in 30 °C for 3 days.

## Additional Information

**How to cite this article**: Lin, Q. *et al.* Robust orthogonal recombination system for versatile genomic elements rearrangement in yeast *Saccharomyces Cerevisiae. Sci. Rep.* **5**, 15249; doi: 10.1038/srep15249 (2015).

## Supplementary Material

Supplementary Information

## References

[b1] Carr P. A. & Church G. M. Genome engineering. Nature biotechnology 27, 1151–1162 (2009). 10.1038/nbt.1590 20010598

[b2] Shao Z. & Zhao H. Construction and engineering of large biochemical pathways via DNA assembler. Methods Mol Biol 1073, 85–106 (2013). 23996442 10.1007/978-1-62703-625-2_9 PMC4321867

[b3] Gibson D. G. *et al.* Creation of a bacterial cell controlled by a chemically synthesized genome. Science 329, 52–56 (2010). 20488990 10.1126/science.1190719

[b4] Henry C., Overbeek R. & Stevens R. L. Building the blueprint of life. Biotechnology journal 5, 695–704 (2010). 20665643 10.1002/biot.201000076

[b5] Isaacs F. J. *et al.* Precise manipulation of chromosomes *in vivo* enables genome-wide codon replacement. Science 333, 348–353 (2011). 21764749 10.1126/science.1205822 PMC5472332

[b6] Giovannoni S. J. *et al.* Genome streamlining in a cosmopolitan oceanic bacterium. Science 309, 1242–1245 (2005). 16109880 10.1126/science.1114057

[b7] Swan B. K. *et al.* Prevalent genome streamlining and latitudinal divergence of planktonic bacteria in the surface ocean. Proceedings of the National Academy of Sciences of the United States of America 110, 11463–11468 (2013). 23801761 10.1073/pnas.1304246110 PMC3710821

[b8] Shao Z. & Zhao H. DNA assembler, an *in vivo* genetic method for rapid construction of biochemical pathways. Nucleic acids research 37, e16 (2009). 19074487 10.1093/nar/gkn991 PMC2632897

[b9] Gibson D. G. *et al.* Complete chemical synthesis, assembly, and cloning of a Mycoplasma genitalium genome. Science 319, 1215–1220 (2008). 18218864 10.1126/science.1151721

[b10] Brown W. R., Lee N. C., Xu Z. & Smith M. C. Serine recombinases as tools for genome engineering. Methods 53, 372–379 (2011). 21195181 10.1016/j.ymeth.2010.12.031

[b11] Grindley N. D., Whiteson K. L. & Rice P. A. Mechanisms of site-specific recombination. Annual review of biochemistry 75, 567–605 (2006). 10.1146/annurev.biochem.73.011303.073908 16756503

[b12] Dymond J. S. *et al.* Synthetic chromosome arms function in yeast and generate phenotypic diversity by design. Nature 477, 471–476 (2011). 21918511 10.1038/nature10403 PMC3774833

[b13] Annaluru N. *et al.* Total synthesis of a functional designer eukaryotic chromosome. Science 344, 55–58 (2014). 24674868 10.1126/science.1249252 PMC4033833

[b14] Saraf-Levy T. *et al.* Site-specific recombination of asymmetric lox sites mediated by a heterotetrameric Cre recombinase complex. Bioorganic & medicinal chemistry 14, 3081–3089 (2006). 16412655 10.1016/j.bmc.2005.12.016

[b15] Lauth M., Spreafico F., Dethleffsen K. & Meyer M. Stable and efficient cassette exchange under non‐selectable conditions by combined use of two site‐specific recombinases. Nucleic acids research 30, e115–e115 (2002). 12409474 10.1093/nar/gnf114 PMC135837

[b16] Guo F., Gopaul D. N. & van Duyne G. D. Structure of Cre recombinase complexed with DNA in a site-specific recombination synapse. Nature 389, 40–46 (1997). 9288963 10.1038/37925

[b17] Dymond J. & Boeke J. The Saccharomyces cerevisiae SCRaMbLE system and genome minimization. Bioengineered bugs 3, 168–171 (2012). 22572789 10.4161/bbug.19543 PMC3370935

[b18] Dujon B. Yeast evolutionary genomics. Nature reviews. Genetics 11, 512–524 (2010). 10.1038/nrg2811 20559329

[b19] Krivoruchko A., Siewers V. & Nielsen J. Opportunities for yeast metabolic engineering: Lessons from synthetic biology. Biotechnology journal 6, 262–276 (2011). 21328545 10.1002/biot.201000308

[b20] Stephanopoulos G. Synthetic biology and metabolic engineering. ACS synthetic biology 1, 514–525 (2012). 23656228 10.1021/sb300094q

[b21] Cheng A. A. & Lu T. K. Synthetic biology: an emerging engineering discipline. Annual review of biomedical engineering 14, 155–178 (2012). 10.1146/annurev-bioeng-071811-150118 22577777

[b22] Minorikawa S. & Nakayama M. Recombinase-mediated cassette exchange (RMCE) and BAC engineering via VCre/VloxP and SCre/SloxP systems. BioTechniques 50, 235–246 (2011). 21548907 10.2144/000113649

[b23] Suzuki E. & Nakayama M. VCre/VloxP and SCre/SloxP: new site-specific recombination systems for genome engineering. Nucleic acids research 39, e49 (2011). 21288882 10.1093/nar/gkq1280 PMC3082901

[b24] Karimova M. *et al.* Vika/vox, a novel efficient and specific Cre/loxP-like site-specific recombination system. Nucleic acids research 41, e37 (2013). 23143104 10.1093/nar/gks1037 PMC3553980

[b25] Hoess R. H., Wierzbicki A. & Abremski K. The role of the loxP spacer region in P1 site-specific recombination. Nucleic acids research 14, 2287–2300 (1986). 3457367 10.1093/nar/14.5.2287 PMC339658

