# Supplementary material for: Robust orthogonal recombination system for versatile genomic elements rearrangement in yeast Saccharomyces Cerevisiae
Source: Sci Rep. 2015 Oct 19;5:15249. doi: 10.1038/srep15249 (PMC4609996; doi:10.1038/srep15249)
Supplement: Supplementary Information [file srep15249-s1.pdf]

Robust orthogonal recombination system for versatile genomic elements rearrangement in yeast *Saccharomyces Cerevisiae*

Qiuhui Lin[a,b] *, Hao Qi[a,b] *, Yi Wu[a,b], Yingjin Yuan[a,b] †

a: Key Laboratory of Systems Bioengineering (Ministry of Education), Tianjin University, Tianjin, 300072, PR China

b: SynBio Research Platform, Collaborative Innovation Center of Chemical Science and Engineering (Tianjin), School of Chemical Engineering and Technology, Tianjin University, Tianjin, 300072, PR China

* These authors contributed equally to this work.

† Correspondence to: yjyuan@tju.edu.cn

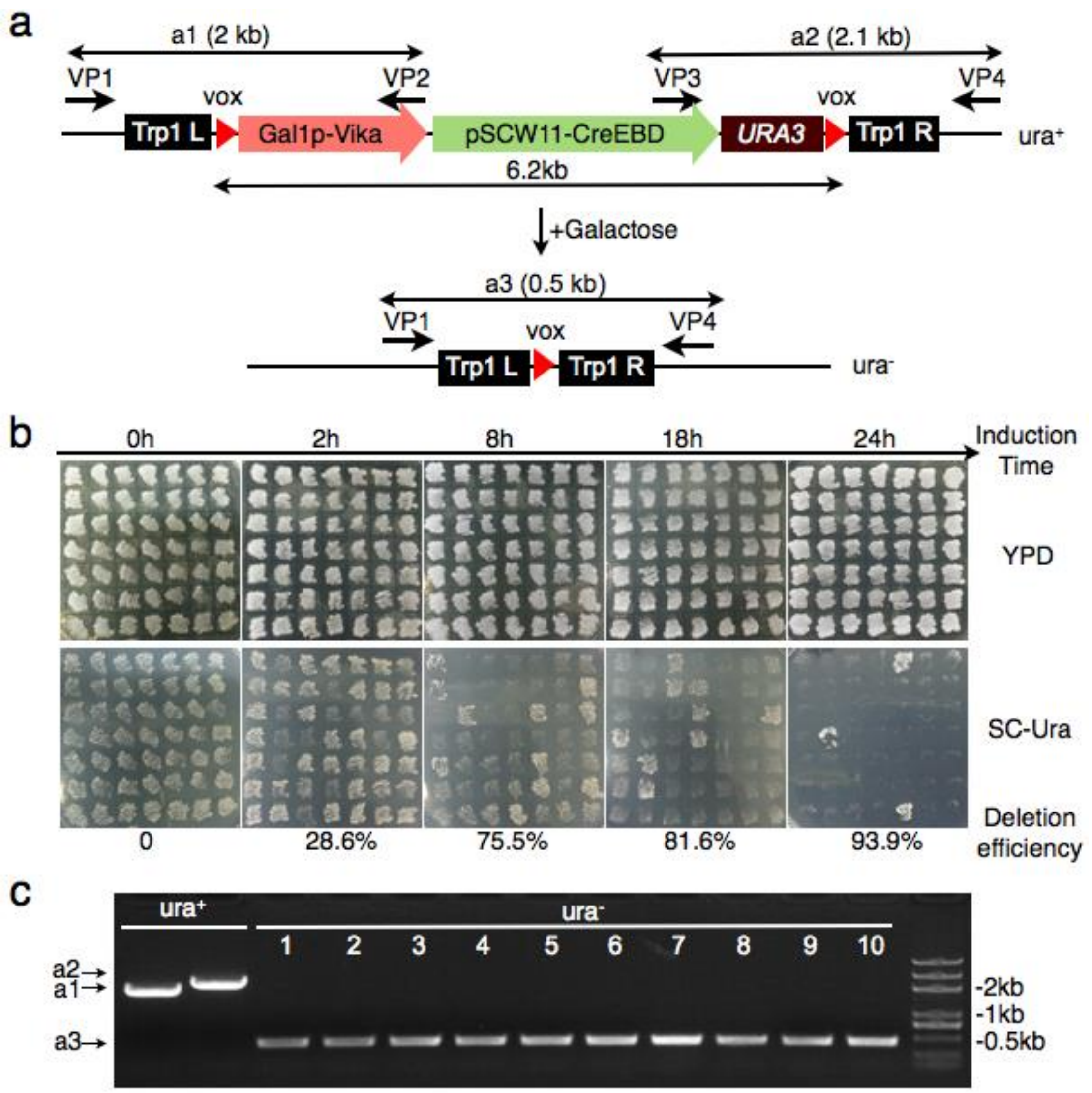

Supplementary Figure 1| Vika mediated DNA cassette self-excision. (A) Schematic of DNA cassette encoding recombinase vika and selective marker URA3 flanked by vox site for self-excision. (B) After inducing expression of recombinase Vika, cell was collected at various time point and grown on non-selective YPD and selective SC-Ura plate. (C) Excision was confirmed by amplified corresponding a3 DNA fragment from cell collected from 10 cell colonies at time point of 24hr. The most left two lanes are DNA fragement a1 and a2 amplified from the cell before Vika-mediated excision.

Table S1 : Strains used in this study

Name	Genotype/Description	Source
**Strain**		
BY4741	MATa (Leu2Δ0 LYS2 met15 his3Δ1 ura3Δ0)	From Prof. Jef Boeke[1]
BY4742	MATα (his3△1, leu△0, lys2△0, ura3△0)	From Prof. Jef Boeke[1]
synIII	MATα (MET15 lys1Δ0 ura3Δ0 his3Δ1leu2Δ0 synIII sup61::HO)	From Porf. Jef Boeke[1]
yLQH201	MATa (Leu2Δ0 LYS2 met15 his3Δ1 ura3Δ0) [pRS415-GAL1p-Cre-loxP-His-loxP]	This study
yLQH202	MATa (Leu2Δ0 LYS2 met15 his3Δ1 ura3Δ0) [pRS415-GAL1p-Cre-vox-Ura3-vox]	This study
yLQH203	MATa (Leu2Δ0 LYS2 met15 his3Δ1 ura3Δ0) [pRS415-GAL1p-Vika-loxP-His3-loxP]	This study
yLQH204	MATa (Leu2Δ0 LYS2 met15 his3Δ1 ura3Δ0) [pRS415-GAL1p-Vika-vox-Ura3-vox]	This study
yLQH205	MATα (MET15 lys1Δ0 ura3Δ0 his3Δ1leu2Δ0 synIII sup61::HO trp1::pSCW11-Cre-pGAL1-Vika-Ura3)	This study
yLQH206	MATα (MET15 lys1Δ0 ura3Δ0 his3Δ1leu2Δ0 synIII sup61::HO trp1::vox-pSCW11-Cre-Ura3-vox)	This study
yLQH207	MATα (MET15 lys1Δ0 ura3Δ0 his3Δ1leu2Δ0 trp1::vox-pSCW11-Cre-pGAL1-Vika-ura3-vox	This study
yLQH208	MATα (MET15 lys1Δ0 ura3Δ0 his3Δ1leu2Δ0 synIII sup61::HO trp1::vox)	This study
yLQH211	MATa (Leu2Δ0 LYS2 met15 his3Δ1 ura3Δ0 HO::vox-RFP-vox-loxP-GFP-loxP-URA3)	This study
yLQH212	MATa (Leu2Δ0 LYS2 met15 his3Δ1 ura3Δ0 HO::vox-RFP-loxP-vox-GFP-loxP-URA3)	This study
**Plasmids**		
pLM158	pSCW11-CreEBD	This study
pUC57-Vika	Codon-optimized Vika ORF	Genewiz. Inc
pRS vector	pRS413, pRS414, pRS415, pRS416	From Prof.
pYES2	Vector with GAL1p and CYC1t	Gift from Prof. Jef Boeke
pLQH122	pYES2-GAL1p-Cre-CYC1t	This study
pLQH124	pYES2-GAL1p-Vika-CYC1t	This study
pLQH134	pRS415-pGAL1p-Cre-CYC1t	This study
pLQH135	pRS415-pGAL1p-Vika-CYC1t	This study

Table S2: Primers used in this study

Primers	Sequence(5'-3')
**Expression plasmid construction**	
Cre-HindIII-F	TATTAAGCTTATGTCCAATTTACTGACCGT
Cre-XbaI-R	GCCCTCTAGATTAATCGCCATCTTCCAGCAGG
Vika-HindIII-F	CGCAAGCTTATGACTGATTTGACTCCTTTCC
Vika-XbaI-R	CGGATCTAGATTATCTTTGTCTCCTCTTCTGA
Gal1p-EcoRI-Trp1-F	AGACGTATTGTCGACCCGGGAATTCCGGATTAGAAGCCGCCGAGC
CYC1t-EcoRI-Trp1-R	ACAAGTAATTGGGCTGCAGGAATTCGCAAATTAAAGCCTTCGAGC
**Orthogonal plasmids construction**	
pRS415-XhoI-GAL1p-F	ATTGGGTACCGGGCCCCCCCTCGAGCGGATTAGAAGCCGCCGAGC
CYC1t-vox-R	**AATACGTCTGAGAATGGGCGTTCTCAGACCTATT**GCAAATTAAAGCCTTCGAGC
CYC1t-loxP-R	**ATAACTTCGTATAATGTATGCTATACGAAGTTAT**GCAAATTAAAGCCTTCGAGC
vox-Ura3-F	**AATAGGTCTGAGAACGCCCATTCTCAGACGTATT**TTCAATTCATCATTTTTTTT
Ura-vox-R1	**AATACGTCTGAGAATGGGCGTTCTCAGACCTATT**TTAGTTTTGCTGGCCGCATC
Ura-vox-pRS415-XhoI-R2	GGAATTCGATATCAAGCTTATCGATACCGTCGACCTCGAGAATACGAATACGTCTGAGAATGGGCG
loxP-His3-F	**ATAACTTCGTATAGCATACATTATACGAAGTTAT**CTAGTACACTCTATATTTTT
His-loxP-R1	**ATAACTTCGTATAATGTATGCTATACGAAGTTAT**CTACATAAGAACACCTTTGG
His-loxP-pRS415-XhoI-R2	GGAATTCGATATCAAGCTTATCGATACCGTCGACCTCGAGAATACGATAACTTCGTATAATGTATG
**Sequential and selective deletion experiment**	
EcoRI-vox-RFP-F	CATGAATTC**AATAGGTCTGAGAACGCCCATTCTCAGACGTATT**AGCTTTTCAATTCAATTCATCA
RFP-vox-KpnI-R	TATGGTACC**AATACGTCTGAGAATGGGCGTTCTCAGACCTATT**TCAATAAGAGCGACCTCAT
RFP-loxP-KpnI-R	ATTGGTACC**ATAACTTCGTATAATGTATGCTATACGAAGTTAT**TCAATAAGAGCGACCTCATG
KpnI-vox-GFP-F	TATGGTACC**AATAGGTCTGAGAACGCCCATTCTCAGACGTATT**TGTTTGCAAAAAGAACAAAAC

KpnI-loxP-GFP-F	TATGGTACC**AATACGTCTGAGAATGGGCGTTCTCAGACCTATT**TCAATAAGAGCGACCTCAT
GFP-loxP-BamHI-R	TTGGGATCC**ATAACTTCGTATAATGTATGCTATACGAAGTTAT**AAAGCCACGCGTGTGCACC
**SCRaMbLE termination experiment**	
trp1L-vox-F	ATGTCTGTTATTAATTTCAC
trp1L-vox-R	ggcggcttctaatccg**AATACGTCTGAGAATGGGCGTTCTCAGACCTATT**AGGAACTCTTGGTATTCTT
GAL1p-Vika-CYC1t-F	aataccaagagttcct**AATAGGTCTGAGAACGCCCATTCTCAGACGTATT**CGGATTAGAAGCCGCCGAG
GAL1p-Vika-CYC1t-R	aagaatcacctctgggtagtagtatctcattgcgtcaagacacaagtaatGCAAATTAAAGCCTTCGAG
pSCW11-CreEBD-F	tgaaaaccttgcttgagaaggttttgggacgctcgaaggctttaatttgcAATTACTTGTGTCTTGACG
pSCW11-CreEBD-R	caaagaaaccgaaatcaaaaaaaagaataaaaaaaaaatgatgaattgaaTCAAGCAAGGTTTTCAGTA
URA3-vox-F	ctgtacagacgcgtgtacgcatgtaacattatactgaaaaccttgcttgaTTCAATTCATCATTTTTTT
URA3-vox-R	aataactggcaaaccg**AATACGTCTGAGAATGGGCGTTCTCAGACCTATT**TTAGTTTTGCTGGCCGCAT
pSCW11-Cre-F	ATTCTCAGACGTATTGTCGACCCGGGAATTCctgcagcccAATTACTTGTGTCTTGACG
pSCW11-Cre-R	AAAGAATAAAAAAAAAATGATGAATTGAATTGAAATCGATTCAAGCAAGGTTTTCAGTA
Ura3-Cre-F	TGTAACATTATACTGAAAACCTTGCTTGAATCGATTTCAATTCAATTCATCATTTTTTT
Ura-vox-R	AATAACTGGCAAACCGAATACGTCTGAGAATGGGCGTTCTCAGACCTATTttagttttgctggccgcat
vox-Trp1R-F	cggccagcaaaactaaAATAGGTCTGAGAACGCCCATTCTCAGACGTATTCGGTTTGCCAGTTATTAAA
vox-Trp1R-R	CTATTTCTTAGCATTTTTGACGAAA
VP1	GCACGTGAGTATACGTGATT
VP2	GCAAATTAAAGCCTTCGAGC
VP3	AAACGCTCTAAGGAGAACA
VP4	CTTGCTTTTCAAAAGGCCTG

References:

1. Annaluru, N. et al. Total synthesis of a functional designer eukaryotic chromosome. *Science* **344**, 55-58 (2014).
